# Inhibition of mesenchymal stromal cells by pre-activated lymphocytes and their culture media

**DOI:** 10.1186/scrt392

**Published:** 2014-01-09

**Authors:** Erica Valencic, Claudia Loganes, Stefania Cesana, Elisa Piscianz, Giuseppe Gaipa, Ettore Biagi, Alberto Tommasini

**Affiliations:** 1 Department of Pediatrics, Institute of Maternal and Child Health IRCCS Burlo Garofolo, Via dell’Istria 65/1, 34137 Trieste, Italy; 2 M. Tettamanti Research Center, Pediatric Clinic University of Milano Bicocca, Monza, Italy; 3 Cell Therapy Laboratory “Stefano Verri”, San Gerardo Hospital, Monza, Italy

## Abstract

**Introduction:**

Despite having a proven immunosuppressive potential *in vitro*, human mesenchymal stromal cells (MSCs) are reported to display variable efficacy *in vivo* and, in fact, their proven benefit in the clinical practice is still limited and controversial.

**Methods:**

The interplay between clinical grade MSCs and pre-activated donor lymphocytes or selected lymphocyte subsets was studied *in vitro*. The kinetics of MSC growth and viability was evaluated by adhesion-dependent changes of culture plate impedance and biochemically by a colorimetric assay. Activation of natural killer (NK) cells was assessed as well, using a flow cytometry assay.

**Results:**

A strong inhibition of MSC growth was rapidly induced by the addition of pre-activated lymphocytes but not of resting lymphocytes. Inhibition seems not to be attributable to a single cell population, as similar results can be obtained by depleting NK cells or by using either selected CD4^+^ or CD8^+^ lymphocytes. In addition, conditioned medium (CM) from activated lymphocytes was able to inhibit MSC growth in a dose-dependent manner. Furthermore, licensing with IFN-γ partially protected MSCs from pre-activated lymphocytes but not from their CM. These results suggest an inhibitory role of lymphocyte-activation-derived substances. However, the identification of a single molecule responsible for MSC inhibition remained elusive, even if preliminary experiments showed that ATP and, to a lesser extent, TNF-α might play a role.

**Conclusions:**

These results suggest that survival of MSCs can be affected by soluble mediators released by activated lymphocytes. Thus it can be hypothesized that MSC immunosuppressive action *in vivo* could be impaired by ongoing immune activation through the release of inflammatory mediators.

## Introduction

Mesenchymal stromal cells (MSCs) are a heterogeneous population of cells that can be obtained from bone marrow and from other adult tissues and are able to proliferate *in vitro* as plastic-adherent fibroblast-like cells [1-7]. *In vitro*, they have a potent immunosuppressive action that justified their use in clinical trials for the treatment of steroid-resistant graft versus host disease (GvHD) and for severe autoimmune diseases. In fact, MSCs are thought to be poorly immunogenic and have been shown to have a highly safe profile *in vivo*. However, the results of clinical trials so far showed heterogeneous efficacy [8-11], and contradictory results have been published about their immunogenicity [12-14]. In particular, it is becoming evident that microenvironmental factors may play a major role in conditioning the immunosuppressive action of MSCs. On one hand, natural killer (NK) cells are able to kill MSCs in appropriate conditions [15]. On the other hand, the immunosuppressive potency of MSCs could be increased *in vitro* by their exposure to exogenous molecules, such as lymphocyte-derived cytokines, such as interferon-gamma (IFN-γ). For example, MSCs that have been previously exposed *in vitro* to IFN-γ are resistant to NK-cell-mediated lysis, maybe due to an up-regulation of class 1 human leukocyte antigen (HLA-I) on their surface [15,16]. As a matter of fact, pre-clinical results may vary substantially according to the setting of the co-culture between lymphocytes and MSCs. We have previously shown that whether lymphocytes are activated before or during incubation with MSCs and, the other way around, whether MSCs are treated or not with licensing-substances before the co-culture are both critical factors influencing the outcome of immunosuppression.

In our model, the stimulation of lymphocytes before incubation with MSCs is meant to mimic the GvHD condition *in vivo*, considering that the therapeutic use of MSCs is proposed for patients with already active immune responses. In this model, pre-activated lymphocytes are resistant to the action of MSCs, unless MSCs have been previously exposed to IFN-γ [17].

In the present study, to unravel better the interactions between lymphocytes and MSCs, we used a non-invasive tool for real-time monitoring of the vitality and growth of MSCs after the addition of peripheral blood mononuclear cells (PBMCs) [18].

## Methods

### Cell culture

Human bone marrow MSCs were obtained at the Laboratory for Cell and Gene Therapy ‘S Verri’, Monza, Italy [11]. Side collections of clinical grade MSC preparations were dedicated to this research, after obtaining the donors’ informed consent. The research plan was approved by the Institute Research Board (reference number #RC 32/11) and by the local ethics committee at the IRCCS Burlo Garofolo, Trieste, Italy.

In brief, total nucleated cells were isolated from the washouts of discarded bone marrow collection bags and filters by washing both the filter and the empty bag twice with phosphate-buffered saline and centrifuging at 1,600 rpm for 5 minutes. The cells were plated at 500,000 cells/cm^2^ in Dulbecco’s modified essential medium low glucose (Gibco-Invitrogen, Carlbad, CA, USA), supplemented with 2 UI/ml heparin (Pharmatex, Milan, Italy) and 5% platelet lysate (hPL) and cultured in 5% CO_2_ at 37°C. Non-adherent cells were removed after two to three days and a 50% medium change was performed twice weekly until 80% confluence. Cells were recovered by trypsinization, re-plated at 200 cells/cm^2^ and cultured for an additional 12 days. After trypsinization, the cells were frozen in vials in 10% clinical grade dimethyl sulfoxide (DMSO) (WAK-Chemie Medica GmbH, Steinbach, Germany) and 90% human AB plasma.

When ready to be used, the cells were thawed and plated in T75 flask (Falcon, BD Biosciences, Bedford, MA USA) at 8,000 cells/cm^2^ in Dulbecco’s modified essential medium low glucose (Euroclone, Milan, Italy), supplemented with 100 U/ml penicillin, 100 μg/ml streptomycin, 200 mM L-glutamine (Euroclone), 5% platelet lysate (called complete medium), and cultured in 5% CO_2_ at 37°C. Following trypsinization, at the third passage, the cells were seeded in a 16-well electric microtiter plate for the co-culture experiments.

PBMCs were isolated from adult healthy volunteers, after obtaining their informed consent according to the local ethical committee, by centrifugation on biocoll separating solution (Biochrom AG, Berlin, Germany) at 560 × g for 30 minutes at room temperature. CD4^+^ and CD8^+^ cells were then separated by negative selection using magnetic sorting (Miltenyi Biotec, Bergisch Gladbach, Germany). PBMCs depleted of CD56^+^ cells were obtained using anti-CD56 coated beads (Miltenyi Biotec).

PBMC, CD4^+^ cells, CD8^+^ cells, CD56-depleted PBMCs were stimulated or not with 1 μg/ml phytohemagglutinin (PHA) (Sigma Aldrich, Milan, Italy) 24 hours before the co-culture and used as effector cells to test how they affect MSC growth.

In a new set of experiments, MSCs were cultivated in the presence of conditioned medium (CM) from 1 × 10^6^ PBMCs activated with 1 μg/ml PHA or with anti CD3/CD28 beads (Dynabeads® Human T-activator CD3/CD28, Invitrogen Dynal AS, Oslo, Norway) in complete medium for 24 hours.

CM from PBMCs, activated or not with PHA, were assessed by means of a bead-based multiplex immunoassay (27 human-Bio-Plex assay; BioRad Laboratories, Milan, Italy), following the manufacturer’s instructions. Human cytokine and chemokine levels were measured in duplicate, using the Bio-Plex 200 reader (Bio-Rad, Hercules, CA, USA) equipped with Bioplex Manager 6 software.

### Co-culture experiments with impedance measurements

Measurements were carried out with an xCELLigence RTCA DP instrument (Roche, Penzberg, Germany). In this instrument, the presence of adherent cells on the bottom of the plate leads to an increase of electrode impedance, which is converted in a ‘Cell Index’ (CI) that depends on the number and viability of attached cells. Thus, the death and detachment of cultured cells will be recorded as a decrease in impedance. In this 16-well device (E-Plate 16) 400 MSCs/wells were plated in 200 μl of complete medium and the impedance was measured every five minutes.

Cells were allowed to attach and proliferate. When they had reached their growth phase, as evidenced by curve profiles, 100 μl of medium were removed and PBMCs were added at varying cell ratios to seeded MSCs or CM was added at varying dilutions.

In a set of experiments, attached MSCs were pre-treated with IFN-γ (at a final concentration of 1,000 U/ml), 24 hours before the stimulation.

This impedance assay was reproduced four times with different MSC populations, treated with PBMCs (or their CM) from various healthy volunteers. Each experiment used a single donor’s cells and was conducted in triplicate.

### NK degranulation assay

The ability of MSCs to stimulate NK cells was investigated by flow cytometry, by measuring the MSC-induced NK-degranulation as revealed by CD107a surface expression [19]. Briefly, 2 × 10^5^ donor PBMCs (resting or PHA-activated) and 2 × 10^5^ MSCs were co-cultured in suspension in the presence of anti-CD107a-PE (BD Biosciences, San Jose, CA, USA). An equal number of PBMCs was incubated with 2 × 10^5^ K562 tumor cell line (ATCC CCL 243), as positive control of degranulation, or with medium alone (Iscove’s modified Dulbecco’s medium, IMDM), as negative control. After two hours of incubation at 37°C and 5% CO_2_ cells were harvested and stained with CD3-PerCP and CD56 APC (both from BD Biosciences). Data were acquired on a CyAn ADP flow cytometer (Beckman Coulter, Fort Collins, CO, USA) and analyzed using FlowJo software v 7.6 (TreeStar, Ashland, OR, USA). The expression of CD107a was recorded on CD3^-^ CD56^+^ cell gate.

### MTT cell viability assay

Cell viability was evaluated biochemically with the MTT (3-(4,5-dimethylthiazol-2-yl)-2,5-diphenyltetrazolium bromide) method, after MSC treatment with different volumes of resting or pre-activated lymphocyte CM.

The assay was carried out under the same experimental conditions as those adopted for the xCELLigence protocol: 400 MSCs/well were seeded in a 96-well plate in 200 μL of complete medium; cells were allowed to proliferate and were treated, after about 72 hours, with lymphocyte CM at varying dilutions. Control wells were filled with medium alone to provide the blanks for absorbance readings and untreated control cells were included in the assay. To best match the results of xCELLigence kinetics, the MTT assay was repeated at different time points, 24, 48 and 72 hours after the addition of CM; at these time points, 20 μl MTT (5 mg/ml in PBS) were added to each well, including controls, and the culture plate returned to the cell culture incubator for four hours (37°C, 5% CO_2_). Once incubation ended, the medium was carefully removed and 100 μl DMSO were added to dissolve the intracellular punctuate purple precipitate that is proportional to the number of viable living cells. The absorbance was read at 590 nm with a reference filter of 620 nm.

The extent of MTT conversion in treated cells was expressed as a percentage of the viability of the control cells. Each experiment was reproduced three times in duplicate.

### Statistical analysis

Analyses were performed using GraphPad Prism v5c software. Data were evaluated by one-way analysis of variance (ANOVA) and by Student’s t test.

The study was approved by the Institution Research Board and by the Independent Committee for Bioethics of the Institute Burlo Garofolo, with reference number 32/11.

## Results

**Figure 1:**
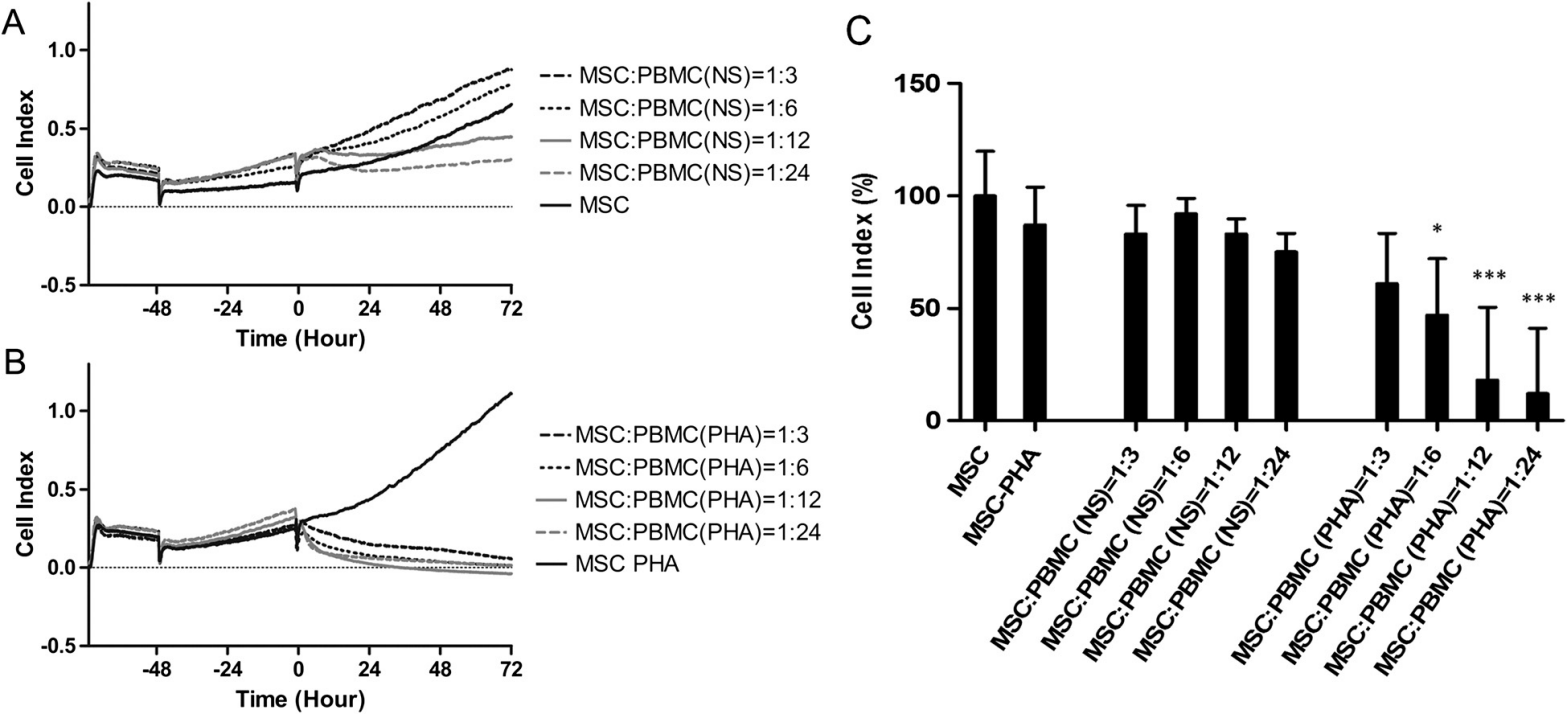
**Impedance profiles and mean CI values of MSCs in co-culture with PBMCs.** Unstimulated (NS) PBMCs **(A)** and PHA-stimulated PBMCs **(B)** were added to MSCs (400/well) after about 72 hours of culture at four different ratios. The experiment was conducted in triplicate. **(C)** The mean CI values, obtained in the four independent experiments, treating MSCs with resting or pre-activated PBMCs. Results were analyzed and expressed as percentage of the untreated MSCs and presented as mean values ± standard deviations. Statistical analysis was performed using one-way ANOVA (*P <0.05; **P <0.01; ***P <0.001). ANOVA, analysis of variance; CI, cell index; MSCs, mesenchymal stem cells; PBMCs, peripheral blood mononuclear cells.

Unstimulated PBMCs had little, if any, effect on the MSC CI. Only at the highest PBMC:MSC ratio (24:1), could a relevant inhibition of MSC growth be observed, as evidenced by a halving of the CI (from a CI of 0.656 to 0.327) (Figure 1A and C).

On the contrary, pre-stimulated PBMCs induced a strong decrease in MSC CI at all ratios, leading in all cases to a complete inhibition of proliferation (CI from 1.11 to 0.054 for 1:3 ratio and 0 for higher ratios). The decrease in MSC CI started in less than an hour after the addition of activated lymphocytes and was faster at higher PBMC:MSC ratios. Lower ratios of PBMCs seemed to exert a delayed effect, as a complete inhibition of MSC growth could be reached in two to three days, suggesting that they can continue proliferating and secreting cytokines during the incubation with MSCs (Figure 1B and C).

**Figure 2:**
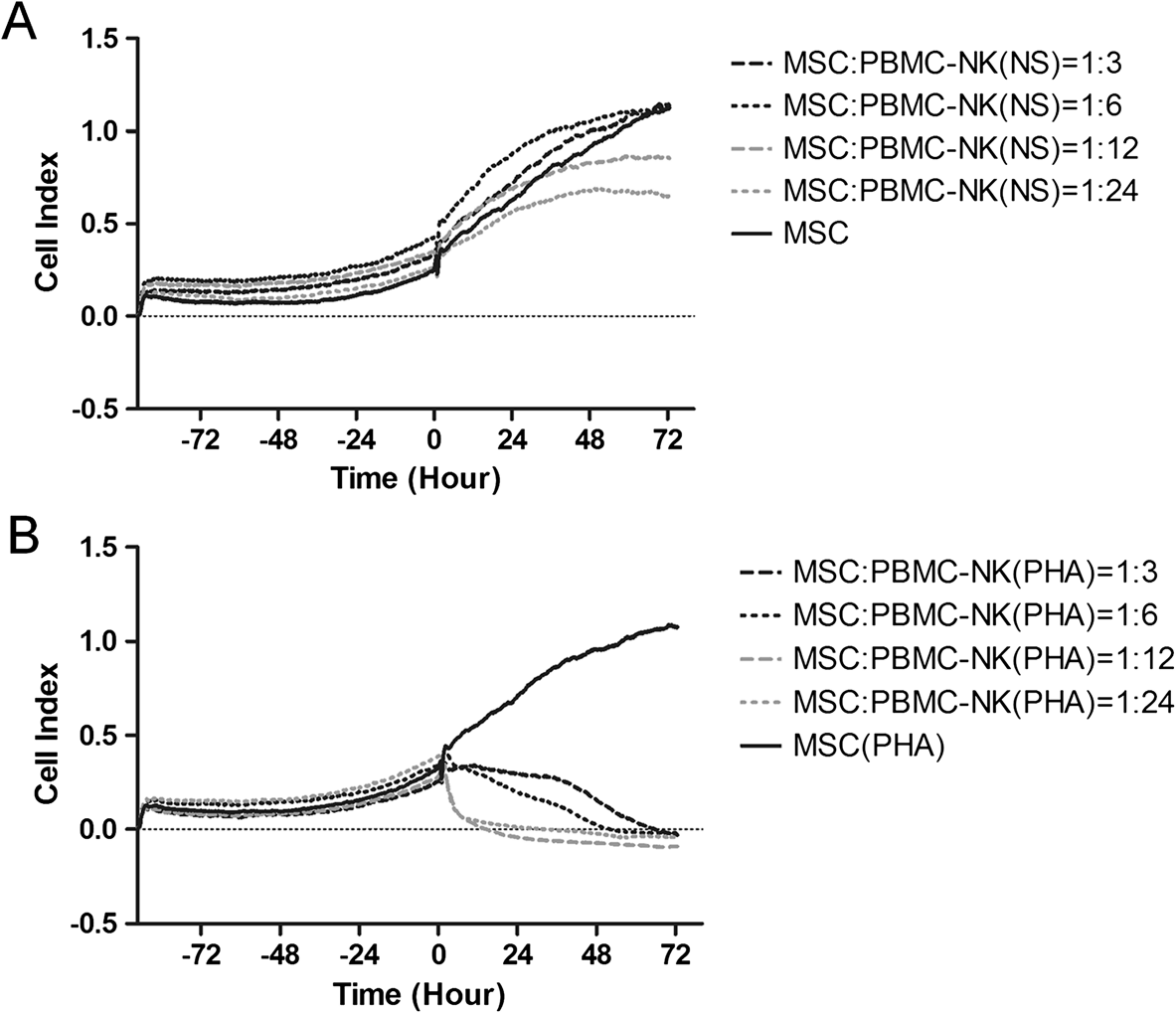
**Impedance profiles of MSCs in co-culture with NK-depleted PBMCs.** Unstimulated (NS) NK-depleted PBMCs **(A)** and PHA-stimulated NK-depleted PBMCs **(B)** were added to MSCs (400/well), after about 72 hours of culture, at four different ratios. The experiment was conducted in triplicate. MSCs, mesenchymal stem cells; NK, natural killer; PBMCs, peripheral blood mononuclear cells; PHA, phytohemagglutinin.

**Figure 3:**
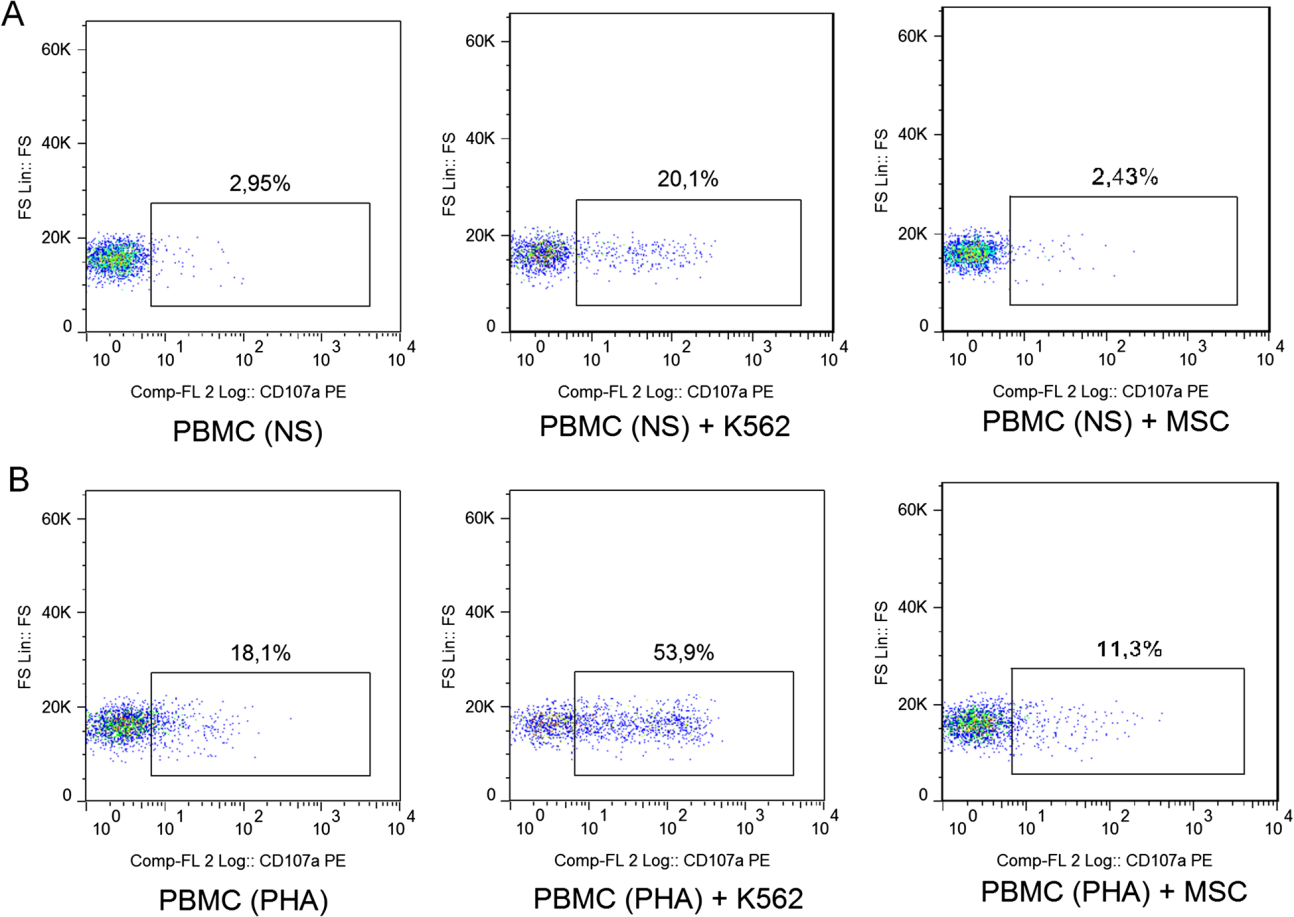
**NK degranulation assay.** Surface expression of CD107a in resting **(A)** or pre-activated **(B)** PBMCs. Three culture conditions were analyzed: PBMCs alone (negative control), PBMCs with the addition of K562 (positive control) and PBMCs with MSCs. For this flow cytometry assay, CD3^-^ CD56^+^ NK cells were gated. MSCs, mesenchymal stem cells; NK, natural killer, PBMCs, peripheral blood mononuclear cells.

The inhibitory capacity of PBMCs on MSCs is retained after depletion of NK cells (Figure 2). This result is in agreement with the observation that MSCs were not able to induce NK activation compared to a conventional tumor cell line, as demonstrated by a CD107a expression similar to the negative control (Figure 3). Furthermore, single lymphocyte subsets (CD4^+^ or CD8^+^) maintained a strong inhibitory capacity on MSCs, which was evident at a 24:1 ratio. However, the effect of CD4^+^ lymphocytes seemed to be more rapid if compared to CD8^+^ cells (data not shown).

**Figure 4:**
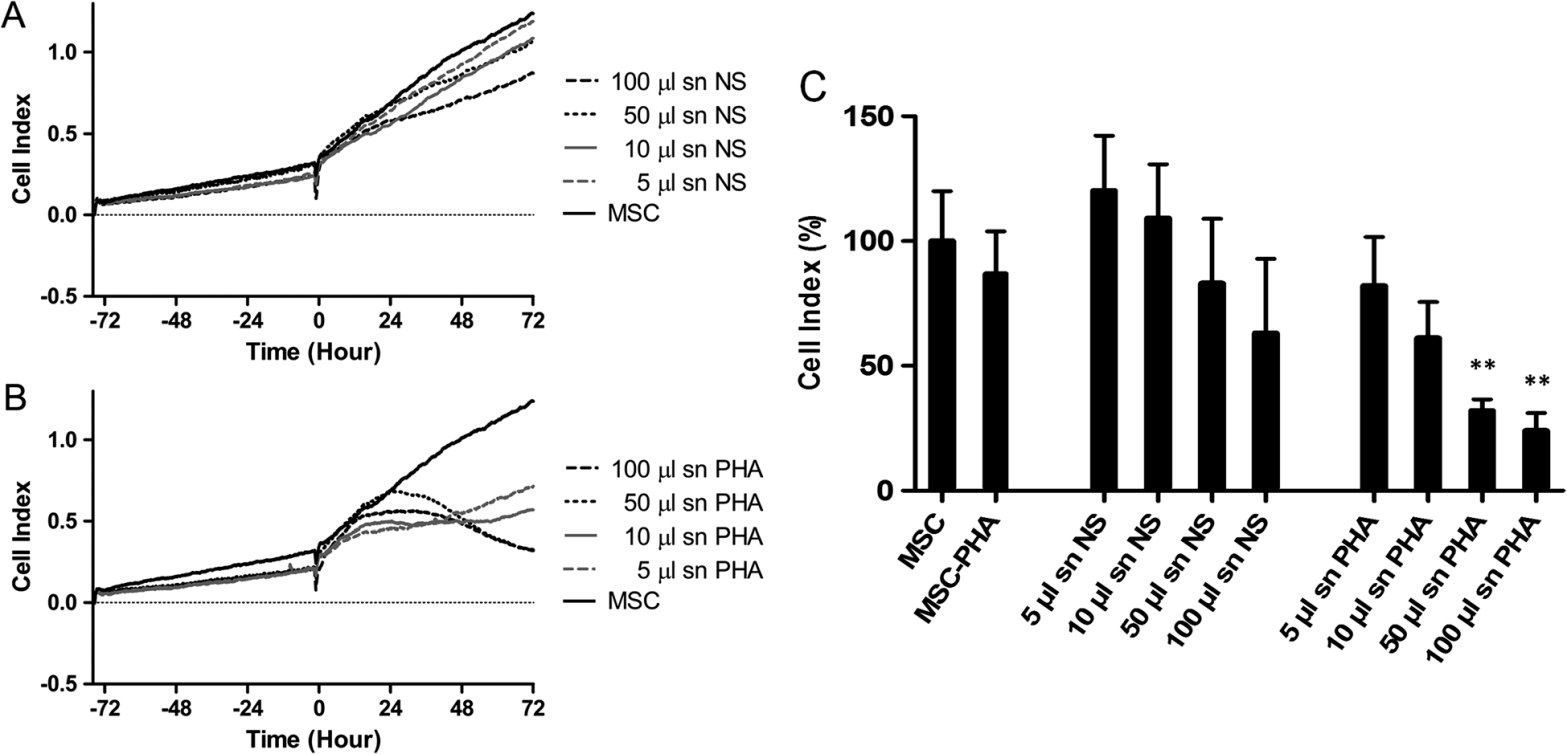
**Impedance profiles and mean CI values of MSCs treated with lymphocyte conditioned medium (CM).** MSCs were stimulated with CM from unstimulated lymphocytes **(A)** and PHA-stimulated lymphocytes **(B)**. The CM was added to MSCs (400/well) at four different dilutions after about 72 hours of culture. The experiment was conducted in triplicate. **(C)** The mean CI values, obtained in the four independent experiments, treating MSCs with CM from resting or pre-activated PBMCs. Results were analyzed and expressed as percentage of the untreated MSCs and presented as mean values ± standard deviations. Statistical analysis was performed using one-way ANOVA (*P <0.05; **P <0.01). ANOVA, analysis of variance; CI, cell index; MSCs, mesenchymal stem cells; PBMCs, peripheral blood mononuclear cells; PHA, phytohemagglutinin.

A subsequent set of experiments was carried out to analyze the role of lymphocyte-derived soluble mediators on MSC growth. The addition of CM from both PHA-activated lymphocytes and antiCD3/CD28-activated lymphocytes inhibited the growth of MSCs in a dose-dependent manner (Figure 4B and C for PHA-activated lymphocytes; Additional file 1: Figure A1 for CD3/CD28-activated lymphocytes). Compared to lymphocytes, CM induced a slower decrease in MSC CI. Moreover, only the highest concentration of CM produced a long lasting inhibition of growth, while a resumption of cell growth occurred during the last day of incubation in the presence of lower concentrations of CM.

**Figure 5:**
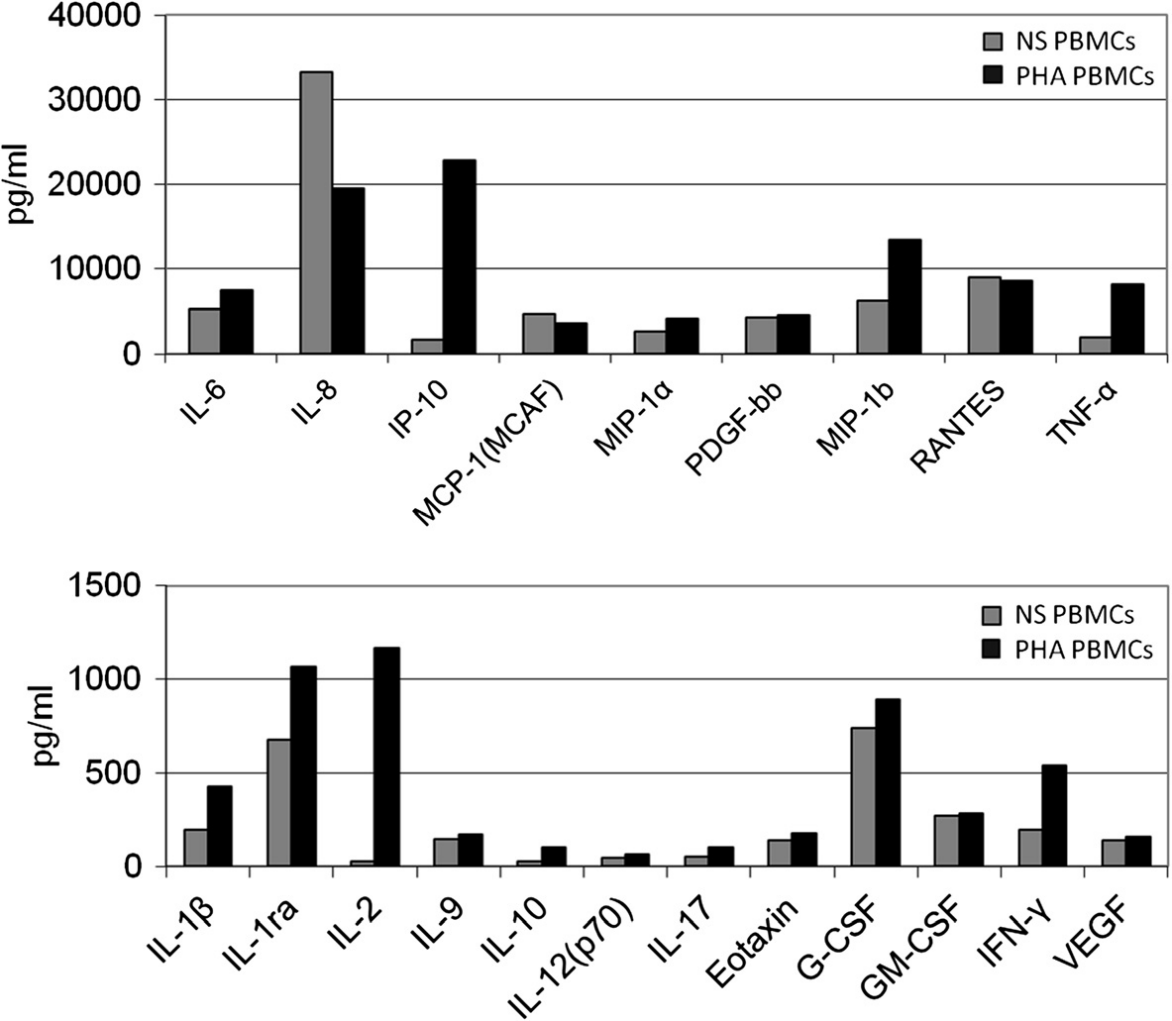
**Measure of different cytokines in the conditioned medium of activated-lymphocytes.** Values are expressed in pg/ml. Non-stimulated lymphocytes: grey bars; lymphocytes stimulated for 24 hours with 1 ug/ml PHA: black bars. The cytokine quantification was conducted in duplicate. PHA, phytohemagglutinin.

Cytokine concentration was assessed in PHA-activated lymphocyte CM, showing high levels of macrophage inflammatory protein-1β (MIP-1β), interferon-inducible protein-10 (IP-10), TNF-α, IFN-γ and IL-2 (Figure 5).

As we have previously shown, licensed MSCs are partially resistant to the action of pre-activated lymphocytes, probably because they are able to block lymphocyte activation and proliferation before becoming themselves a target of lymphocytes [17]. In contrast, pre-exposure to IFN-γ failed to protect MSCs from the action of CM (see Additional file 1: Figure A2 and A3).

**Figure 6:**
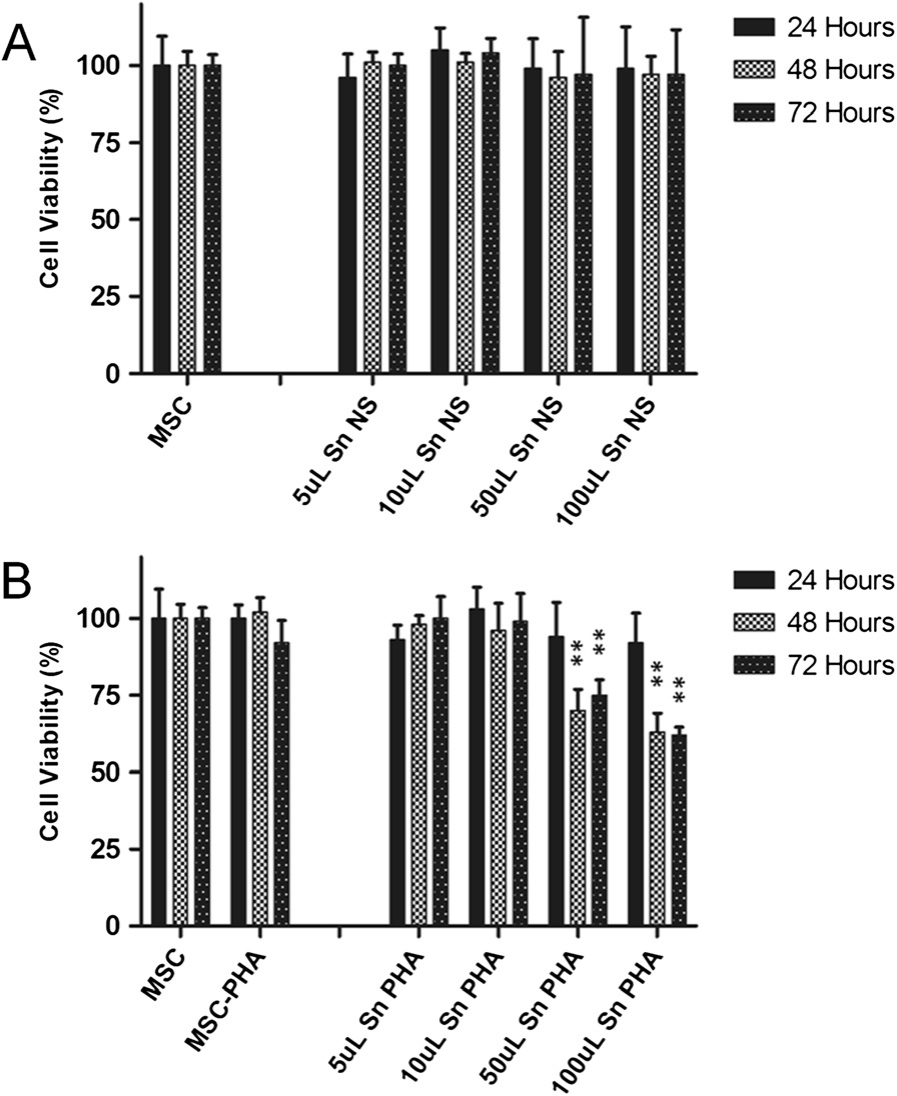
**MTT cell viability assay.** Cell viability was assessed after MSC treatment with the indicated dose of lymphocyte CM. **(A)** CM from unstimulated PBMCs; **(B)** CM from PHA-stimulated PBMCs. Results were analyzed and expressed as percentage of the control (untreated MSCs) and presented as mean values ± standard deviations of the three independent experiments performed in duplicate. Statistical analysis was performed using Student’s t test (*P <0.05; **P <0.01). CM, conditioned medium; MSCs, mesenchymal stem cells; MTT, (3-(4,5-dimethylthiazol-2-yl)-2,5-diphenyltetrazolium bromide; PBMCs, peripheral blood mononuclear cells; PHA, phytohemagglutinin.

The diminution of MSC CI after the addition of lymphocyte CM was paralleled by a diminution in the number of viable cells as assessed by the MTT assay; in particular, the experiment revealed that CM of pre-activated lymphocytes led to a progressive dose-dependent reduction in MSC viability, that was statistically significant only at the higher dose of CM (50 μl and 100 μl), 48 hours and 72 hours post-stimulation (Figure 6).

## Discussion

Although the immunosuppressive potential of MSCs has been consistently demonstrated *in vitro*, the use of these cells in the clinical setting of immune disorders has remained controversial. Few controlled trials have been conducted which showed a highly variable efficacy in different patients [8-10]. The cause of such variability seems not to be attributable to the characteristics of MSCs themselves or to HLA matching between donor and recipient, thus suggesting that the immune activation profiles of each single patient could influence the action of MSCs. Based on these observations, we argued that *in vitro* studies of the immunosuppressive action of MSCs should be carried out by using pre-stimulated lymphocytes, that is, lymphocytes that are already activated when MSCs are added in co-culture. In fact, this condition may better reflect the state of immune activation found in clinical situations, where the therapeutic use of MSCs is proposed [17]. We have previously shown that MSCs are not able to kill pre-stimulated lymphocytes unless they are pre-treated with IFN-γ (17). Here, we analyzed the effect of activated lymphocytes on MSCs by using a new method based on the measure of electric impedance by means of xCELLigence instrumentation, allowing us to calculate a CI that reflects both growth and viability of adherent MSCs. Although plate impedance changes are only indirect measures of cell growth and death, other researchers have shown a very good correlation of impedance data with those obtained with the sulforhodamine colorimetric B assay, which is based on the cellular protein content [20]. Also, in our experience, the impedance changes were paralleled by similar changes in the MTT assay, confirming that in our setting electric impedance correlates well with the number of living cells in the well.

We showed that pre-activated lymphocytes are able to inhibit the growth of bone marrow-derived MSCs. It should be noted that non-stimulated lymphocytes did not significantly affect the growth of MSCs. In contrast, pre-stimulated lymphocytes induced a complete inhibition of MSC growth, even at the lowest lymphocyte:MSC ratios. Moreover, the impedance-based method allowed us to track the kinetics of MSC growth; while higher numbers of lymphocytes produced a dramatic and rapid effect on the growth of MSCs, lower numbers had a slower but progressive effect, which led at the end to the same level of inhibition of MSC growth. This phenomenon probably indicates that stimulated lymphocytes continue their activation and proliferation regardless of MSC presence.

Since it has been shown that MSCs can be recognized and killed by activated NK [15], we hypothesized that the effect of activated lymphocytes on MSCs could be mostly due to the presence of activated NK cells. In fact, we showed that MSCs are not able to trigger NK degranulation compared to a conventional tumor cell line. Moreover, an inhibitory effect on MSCs was maintained by NK-depleted lymphocytes and similar results were obtained as well with purified CD4^+^ or CD8^+^ subsets. Yet, resting lymphocytes had little effect, thus indicating that activation was necessary to induce maximal inhibition of MSC growth. To evaluate whether the inhibitory effect is due to direct contact between MSCs and lymphocytes or to soluble mediators, CM from activated PBMCs was added to MSCs at different dilutions. Indeed, CM inhibited MSC growth in a dose-dependent manner, suggesting that inflammatory cytokines or other mediators released from activated lymphocytes may have a noxious effect on MSCs. Notably, only higher concentrations of CM were able to completely block MSC growth, while only a transient effect was obtained with a progressive dilution of CM. Although the growth of IFN-γ-treated MSCs was only partially affected by pre-activated lymphocytes, it was still inhibited by the addition of CM at certain concentrations. In other words, it is reasonable to state that interferon treatment is effective in potentiating the immunosuppressive action of MSCs, giving them the chance to block the production of lymphocytic mediators that, conversely, can affect the growth of MSCs.

It is noteworthy that a noxious effect of inflammatory cytokines on MSCs has recently been independently demonstrated by different authors. Freytes and collaborators demonstrated that M1 macrophages, producing IL-1β, IL-6, TNF-α and IFN-γ, inhibited the growth of MSC *in vitro* under certain conditions [20]. Furthermore, Liu and collaborators showed that TNF-α and IFN-γ derived from activated lymphocytes are able to block MSC-based bone regeneration [21].

In our setting, TNF-α showed only a limited noxious effect on MSCs when used at supraphysiological concentrations and, thus, it is not likely that it is the main substance responsible for the inhibitory effect of CM. Other biologically active compounds, common to different immune cells, may, of course, play a role. Among these molecules, we could consider nucleotides, such as ATP and ADP, or tryptophan metabolites, known for their immune-regulatory properties. Actually, tryptophan metabolites have been implicated in the immunosuppressive mechanisms of MSCs and, therefore, it is unlikely that they can also affect the viability of MSCs. We, thus, chose to test the action of ATP in our model. We demonstrated that the addition of ATP (to a final concentration of 1 μM and 10 μM) resulted in changes of MSC CI, similar to those obtained with CM derived from pre-activated lymphocytes. This novel finding opened the way to other experimental analysis to be performed to clarify the role of nucleotides on MSCs.

The fact that soluble mediators from activated lymphocytes can affect the growth of MSCs raises concern about the use of MSC-based therapies in conditions characterized by ongoing immune activation. Based on these results, we can assume that MSCs may survive briefly and exert limited effects when infused in patients with strong immune activation. In addition, it could be reasonable to hypothesize that MSCs may last longer when infused as a prophylactic treatment before the occurrence of severe immune activation. In fact, MSCs can survive longer in mice, when infused just after hematopoietic stem cell transplantation (HSCT) [22]. However, several reports suggest a role for stimulated lymphocytes in activating the immunosuppressive properties of MSCs [23-25]. Thus, it seems reasonable to hypothesize that the efficacy of MSC as an immunosuppressant *in vivo* depends upon several factors including: the activation state of lymphocytes, the concentration of inflammatory cytokines in the microenvironment; the ratio between MSCs and lymphocytes; and the licensing of MSCs [26]. As a consequence, the outcome of the struggle between activated lymphocytes and MSCs *in vivo* cannot be easily predicted by preclinical studies *in vitro* or in animal models.

Further studies should investigate how different lymphocyte mediators can affect MSC growth.

In addition, *in vivo* studies are needed to track the effect of the immune activation status on MSC survival.

## Conclusions

In conclusion, strategies aimed at activating MSCs and neutralizing specific mediators released by activated lymphocytes can offer new perspectives for immunomodulatory cell therapies. However, further studies are needed to understand the real impact of lymphocyte-derived mediators on the survival of MSCs infused *in vivo*.

## Abbreviations

CI: cell index; CM: conditioned medium; DMSO: dimethyl sulfoxide; GvHD: graft versus host disease; HLA-I: class I human leukocyte antigen; HSCT: hematopoietic stem cell transplantation; IFN-γ: interferon-γ; IL: interleuken; MSC: mesenchymal stromal cell; MTT: 3-(4,5-dimethylthiazol-2-yl)-2,5-diphenyltetrazolium bromide; NK: natural killer; PBMC: peripheral blood mononuclear cell; PHA: phytohemagglutinin.

## Competing interests

The authors declare they have no competing interests.

## Authors’ contributions

EV designed the experimental plan, performed analysis of results and wrote the manuscript. CL performed additional experiments to review the manuscript and also contributed to the writing of the revised manuscript. SC produced GMP mesenchymal cells, discussed results and corrected the manuscript draft. EP performed the immunologic analyses, discussed the results and corrected the draft. GG supervised the production of mesenchymal cells and discussed the experimental plan. EB discussed results and conclusions of the work. AT is responsible for the project and supervised the entire work. All authors read and approved the final manuscript.

## Supplementary Material

Additional file 1: Figure A1 Impedance profiles of MSCs (400 cells/well) treated with resting or CD3/28-activated PBMC CM. MSCs were stimulated with CM from unstimulated lymphocytes **(A)** and from lymphocytes stimulated with anti CD3/28 beads **(B)**. The CM was added to MSCs (400/well) at three different dilutions after about 72 hours of culture. The experiment was conducted in triplicate. **Figure A2.** Impedance profile of licensed MSCs in co-culture with PBMCs. Resting **(A)** or PHA-pre-activated **(B)** PBMCs were added at four different ratios to MSCs (400/well) pre-treated with IFN-γ. The experiment was conducted in triplicate. **Figure A3.** Impedance profile of licensed MSCs treated with PBMC CM resting **(A)** or pre-activated **(B)** lymphocyte CM was added at four different dilutions to MSCs (400/well) pre-treated with IFN-γ. The experiment was conducted in triplicate. Click here for file
