# Supplementary figures and images for: Inhibition of mesenchymal stromal cells by pre-activated lymphocytes and their culture media
Source: Stem Cell Res Ther. 2014 Jan 9;5(1):3. doi: 10.1186/scrt392 (PMC4055165; doi:10.1186/scrt392)

Additional Files 1

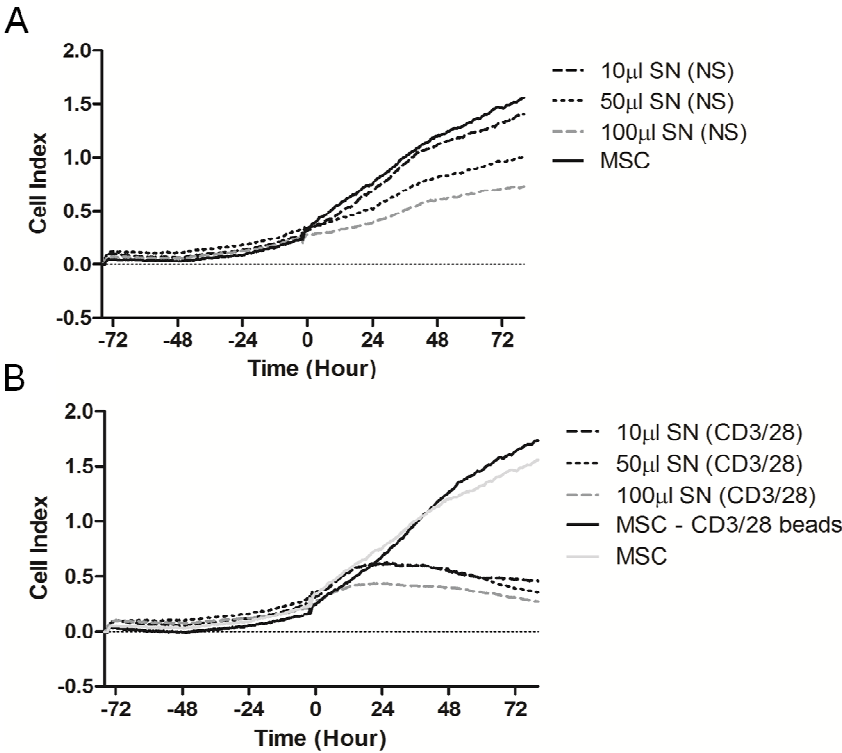

Fig. A1

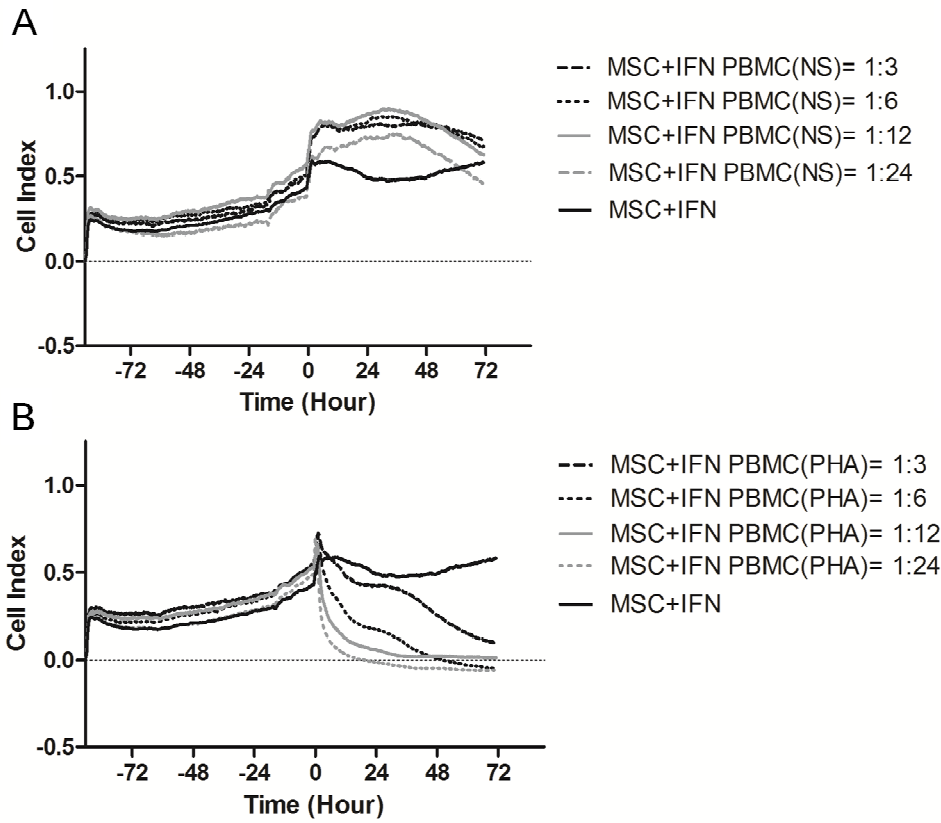

Fig. A2

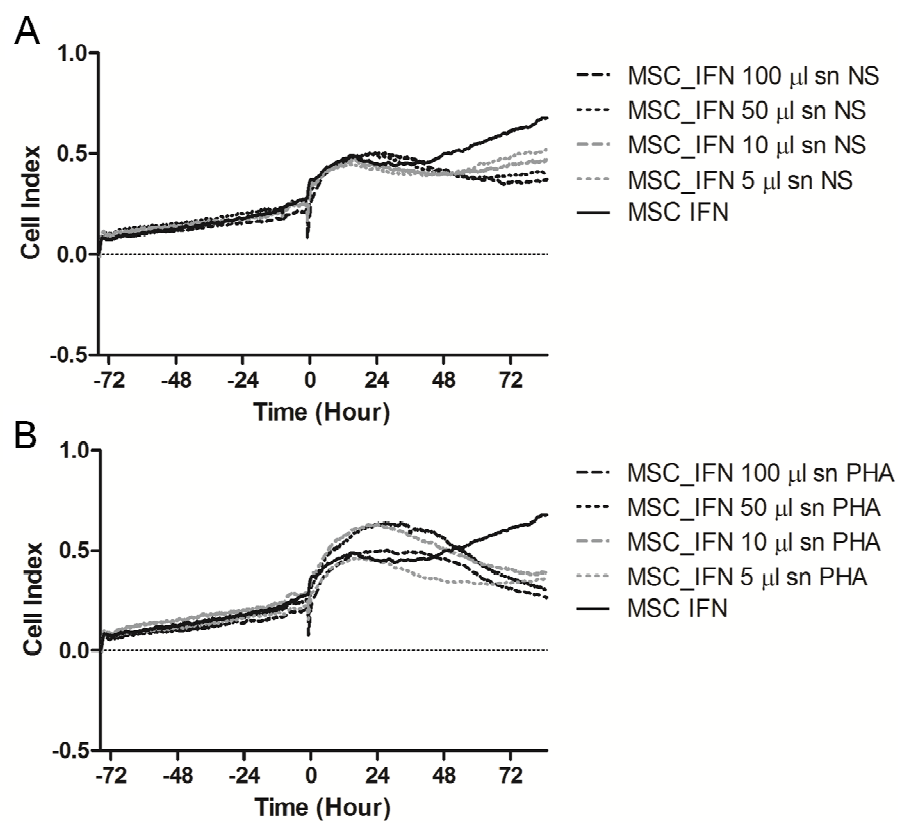

Fig. A3

Supplement: Additional file 1: Figure A1 — Impedance profiles of MSCs (400 cells/well) treated with resting or CD3/28-activated PBMC CM. MSCs were stimulated with CM from unstimulated lymphocytes (A) and from lymphocytes stimulated with anti CD3/28 beads (B). The CM was added to MSCs (400/well) at three different dilutions after about 72 hours of culture. The experiment was conducted in triplicate. Figure A2. Impedance profile of licensed MSCs in co-culture with PBMCs. Resting (A) or PHA-pre-activated (B) PBMCs were added at four different ratios to MSCs (400/well) pre-treated with IFN-γ. The experiment was conducted in triplicate. Figure A3. Impedance profile of licensed MSCs treated with PBMC CM resting (A) or pre-activated (B) lymphocyte CM was added at four different dilutions to MSCs (400/well) pre-treated with IFN-γ. The experiment was conducted in triplicate. [file scrt392-S1.pdf]
